# Referrals for suicidality to a UK child and adolescent mental health service: an evaluation of the policy and practice context of response to suicidal young people

**DOI:** 10.3389/fpsyt.2026.1863579

**Published:** 2026-10-07

**Authors:** Fhionna Moore, Iolanda Salonia, Ayesha Ali, Alissa Mann, Lynne Gilmour

**Affiliations:** 1 National Health Service (NHS) Tayside Child and Adolescent Mental Health Services, National Health Service (NHS) Tayside, Perth, United Kingdom; 2 National Health Service (NHS) Tayside Child and Adolescent Mental Health Services, National Health Service (NHS) Tayside, Dundee, United Kingdom; 3 Department of Veterinary and Medical Sciences, University of Glasgow, Glasgow, United Kingdom; 4 School of Health in Social Science, University of Edinburgh, Edinburgh, United Kingdom

**Keywords:** CAMHS, policy, rejected referrals, service specifications, suicide

## Abstract

**Aims and method:**

We reviewed referrals mentioning suicidality to a Scottish CAMHS. The referrals were from January to June 2019 and 2022. We recorded the referral reason and outcome and compared these to the National CAMHS Service Specification (2020).

**Results:**

Of 2,753 referrals, 560 (20.34%) mentioned suicidality. Of these, 82.4% were accepted. The majority of those rejected were from outside the region, already in the CAMHS, or provided insufficient information. The majority did not include any risk assessment (43.6%) or safety planning (67.7%). We identified lack of clarity in the National CAMHS Service Specification around the requirement for evidence of a serious mental illness and the meaning of “suicidal behaviours”.

**Clinical implications:**

We require clearer language concerning “rejected referrals”, better communication between the CAMHS and referrers, improved pathways for referral re-direction, and support for referrers to assess risk and safety plan. We require clarity in national guidelines over where suicidal young people should be supported.

## Introduction

The leading cause of death for children and young people (CYP) in Scotland in 2011–2020 was probable suicide (1). The Scottish Child and Adolescent Mental Health Services (CAMHS) is governed by the National Service Specification [CNSS (2)], which states that CYP with “suicidal behaviours” are eligible for assessment within the CAMHS (a tier 3 mental health service). The CNSS’ referral criteria specify the following (1): clear symptoms of mental ill health which place the CYP or others at risk and/or are having a significant and persistent impact on day-to-day functioning (including “suicidal behaviours”, which are not defined or described) (2), CYP must be 0–18, and (3) consent must have been given. It is expected that most referrals to the CAMHS will have trialed tiers 1/2 first. Tier 4 services support CYP at immediate risk of harm to themselves or others.

An audit of referrals to two Scottish health boards identified approximately 25% to be for suicidality, with rejection rates ranging from 20% to 70% (3). What remains unknown is whether outcomes were appropriate in the context of the CNSS (2). Were referrals rejected because they did not meet the CNSS referral criteria? Were suicidal CYP who should have been offered timely CAMHS input inappropriately rejected? Answering these questions is essential for the CAMHS to ensure that they are not missing suicidal CYP. Here we reviewed referrals to a Scottish CAMHS to determine (1) whether responses to referrals for suicidality were appropriate in the context of the CNSS, (2) whether suicidal CYP were being missed, and, if so, (3) to identify points of failure.

## Method

### Context

The CAMHS team covers a Scottish health board region situated in Central/East Scotland. It covers an area of nearly 3,000 square miles, with a population of ~417, 650. It includes areas of relatively high population density in two cities and sparsely populated, accessible, and remote rural areas. In relation to provision for suicidal behaviours, the service has a multidisciplinary tier 3 CAMHS team and a tier 4 “CAMHS Response Team” (CRT) which provides crisis support in the community.

### Data sources

Data were collected for two 6-month periods: January 1–June 30, 2019 in order to provide a comparison with (3) and January 1–June 30, 2022 to provide a post-COVID 2019 lockdown comparison.

Incoming referrals are discussed at the weekly “Referral Management Group” (RMG) multidisciplinary team meetings and reviewed against the CNSS (4) to reach one of the following decisions: (1) add to the waiting list for tier 3 assessment, (2) allocate to CRT if more immediate action is required, (3) request further information from the referrer, and (4) redirect to more appropriate services. While some referrals to CRT came via RMG, it was also possible for CRT to receive referrals from other sources (e.g., out of hours). From RMG and CRT records, we extracted the date of birth and the postcode or unique patient number for each referral, allowing us to review digital referrals, decisions, and subsequent actions.

### Data extraction and coding

Suicidality (3): included referrals in their analysis if the primary reason for the referral was suicidality. Here we included all referrals in which suicidality was mentioned, even if not given as the primary reason for referral. This allowed us to investigate outcomes for all referrals that mentioned suicidality with the broadest operationalization and treated a referral as including suicidality however it was described. A subset of 10% of referrals (*n* = 275) were selected at random and double-coded to ensure consistency in coding of suicidality. No discrepancies were noted.

We coded whether suicidality was mentioned in the referral (yes or no) and its severity based on the information included in the referral. Such coding is inherently subjective, and while there is currently no single agreed taxonomy of suicidality, we sought to rank this in light of existing suicide risk models (e.g., Columbia Suicide Severity Scale (4)) as passive ideation (thoughts of suicide), ideation with thoughts about methods (thoughts about suicide that include ideas about how to act on these thoughts), ideation with a concrete plan (thoughts about suicide with a clear plan in mind to make a suicide attempt), and suicide attempt (an attempt to end their life). Examples of information contained in referrals that fell within each severity category are passive ideation —”fleeting thoughts of suicide” or “described suicidal thoughts”, ideation with thoughts about methods—”has thoughts about going to a bridge to end their life and does not plan to act on this”, and ideation with a concrete plan—”had bought paracetamol and hidden in a room with intent to overdose”; suicide attempt was used when the young person was referred following a suicide attempt. A subset of 10% of referrals that mentioned suicidality (*n* = 56) were selected at random and double-coded to ensure consistency in rating of severity. One discrepancy was noted (98% agreement) and inspected, and an agreement was reached.

### Referrer information

We recorded whether the referral included a risk assessment and/or safety plan. Where a referrer had included a comprehensive risk assessment and/or safety plan, we recorded this as “complete”. We considered a comprehensive risk assessment to be a drawing together of information from the CYP that considers risk and protective factors and personal meaning. We considered a comprehensive safety plan to include a delineation of strategies designed to keep the CYP safe based on their personal situation. Where there was evidence that the referrer had discussed risk of suicide and elements of safety planning, we recorded this as “partial”. Where there was no evidence of either, we recorded this as “missing”. A subset of 10% of referrals mentioning suicidality was selected at random and double-coded (*n* = 56). A total of 10 discrepancies were noted in coding of risk assessment (82% agreement) and nine for safety plans (84% agreement). All discrepancies were inspected, and an agreement was reached.

### Demographic characteristics

We recorded age at referral (years) and sex (as recorded in notes, all on the basis of sex allocated at birth).

### CAMHS national service specification referral criteria

To determine whether outcomes of referrals were consistent with the CNSS (2), we compared decisions against the three criteria described in the “Introduction” (i.e., serious mental illness, age of 0–18, and consent provided). We also recorded any alternative reasons given for rejection of referrals as well as the disposal pathways of referrals.

### Consent statement

Following the Scottish Government and National Health Service Research Service (NRS) policy, this audit did not require ethical approval. Clinical governance and Caldicott Guardian approvals were obtained. Consent was not sought as data were extracted from existing records, and the local Caldicott Guardian provided proxy consent, as per NRS policy.

## Results

A total of 3,185 referrals were logged over the two 6-month periods (see **Table 1**). Of these, 432 (13.56%) referrals could not be accessed, meaning that suicidality could not be assessed. Of the remaining 2,753 referrals, 560 referrals for 499 CYP included reference to suicidality (20.34%). The referrals came from GPs (58.84%), schools (19.02%), other NHS services including accident and emergency departments (10.65%), social work (10.42%), and third-sector organizations (1.07%).

**Table 1:** Numbers of referrals to a Scottish CAMHS team in January–June 2019 and January–June 2022, with percentages which mentioned suicidality, percentages at each level of severity of suicidality, and male and female CYP.

	Total		2019^a^	2022	Male	Female
Number of referrals received	3,185		1,439	1,746	1,528	1,651^b^
Number of referrals for which suicidality could be assessed	2,753		1,230	1,523	1,528	1,651
Number of referrals that mentioned suicidality (with percentages in parentheses)	560 (20.34%)		272 (22.11%)	288 (18.91%)	180 (11.78%)	380 (23.03%)
Number of referrals that mentioned suicidality at each level of severity (with percentages in parentheses)	Passive ideation	50 (8.9%)	23 (8.5%)	27 (9.4%)	21 (10.4%)	29 (7.1%)
	Ideation with thoughts about methods	100 (17.9%)	63 (23.2%)	37 (12.8%)	35 (21.5%)	65 (16.4%)
	Ideation with a concrete plan	252 (45%)	118 (43.4%)	134 (46.5%)	81 (49.7%)	171 (43.1%)
	Suicide attempt	112 (20%)	37 (13.6%)	75 (26%)	9 (5.5%)	103 (25.9%)

^a^ Sufficient information to determine severity was missing from 31 referrals for 2019 and 15 for 2022 as well as 34 for men and 12 for women.

^b^ Information on sex was missing from six referrals.

Given the difference in percentages of referrals that mentioned suicidality between 2019 and 2022 and between boys and girls, we carried out unplanned statistical analyses to further explore these. There was a significant association between year and proportion of referrals which mentioned suicidality (Pearson’s chi-square = 4.31, *p* = 0.038); more referrals mentioned suicidality in 2019 (272) than expected (250.2) and fewer in 2022 (*n* = 288) than expected (*n* = 309.8). There was also a significant association between sex and whether suicidality was mentioned in a referral (Pearson’s chi-square = 69.04, Cramer’s *V* = 0.15, *p* < 0.001), with more girls’ referrals mentioning suicidality than expected (380 vs. 290.8) and fewer boys’ referrals than expected (180 vs. 269.2).

1. Were outcomes of referrals that mentioned suicidality appropriate in the context of the CNSS (2020)?

As shown in **Table 2**, approximately 83% of referrals mentioning suicidality were accepted into the CAMHS on a variety of pathways. The remaining 17% were classified as rejected.

**Table 2:** Outcomes of 560 referrals mentioning suicidality in total and for 2019 and 2022 (with percentages in parentheses).

Outcome	Total (%)^a^	2019 (%)	2022 (%)
Offered urgent assessment	305 (54.5%)	159 (58.5%)	146 (50.7%)
Added to waiting list for assessment	148 (26.4%)	59 (21.7%)	89 (30.9%)
Rejected	97 (17.3%)	51 (18.8%)	46 (16%)
Offered CAMHS worker	6 (1.1%)	0	6 (2%)
Consultation	1 (0.2%)	1 (0.4%)	0
Medical review	1 (0.2%)	0	1 (0.3%)

aInformation on outcomes was missing for two referrals.

As shown in **Table 3**, of the rejected referrals, approximately 44% did not meet the CNSS (2020) referral criteria. The remainder were inappropriate referrals as the CYP was either already accepted into the CAMHS or a resident outside of the region or insufficient information was included to make a decision.

2. If referrals for suicidal CYP were inappropriately rejected, what were the points of failure in the referral to treatment pathway that allowed this to happen?

We identified two possible points of failure based on the reasons for rejection listed in **Table 3**. A total of 26 referrals which mentioned suicidality were rejected with the reason “No evidence of serious mental health problems” given. On inspection, all of these cases referenced passive suicidal ideation, and most were redirected to tiers 1 and 2 services, which would appear to be consistent with the CNSS (2020). For eight of these cases, however, no further action (e.g., signposting and re-direction to tiers 1 and 2) was taken. It is possible, then, that a small group of CYP who express passive suicidal ideation not appropriate to specialist tier 3 was not being redirected to appropriate community supports.

**Table 3:** Reasons for rejection of referrals mentioning suicidality and disposal pathways, with percentages in parentheses (reasons for rejection missing from five referrals).

Reason for rejection	*n* (%)	Disposal pathway	*n* (%)
Did not meet the CNSS (2020) referral criteria			
1. No evidence of serious mental health problems which place the CYP or others at risk and/or are having a significant and persistent impact on day-to-day functioning	26 (26.7%)	Directed to school nursing/counsellor	6 (25.1%)
		Directed to community supports	8 (33.4%)
		Directed to “beating the blues” (online NHS CBT programme for low mood)	1 (4.2%)
		Directed to social work	3 (12.5%)
		No action	8 (24.8%)
2. CYP is over 18 or 16 or over and no longer in school	14 (14.4%)	Sent back to referrer for redirection to adult mental health services	13 (92.9%)
		Already in adult mental health services	1 (7.1%)
3. Consent had not given for the referral	3 (3.1%)	Returned to referrer to request consent	3 (100%)
Additional reasons for rejection			
Not resident in the region	3 (3.1%)	Referral returned with request to redirect to appropriate CAMHS team	3 (100%)
CYP already being seen in the CAMHS (i.e., additional referral provided inappropriately and/or submission of additional information by referrer)	15 (15.5%)	Treatment continued	15 (100%)
Already on the CAMHS waiting list	27 (27.8%)	Expedited	2 (7.4%)
		Remain on waiting list	25 (92.6%)
Incomplete referral	4 (4.1%)	Sent back to referrer for further information	4 (100%)

A total of 14 referrals were rejected because the CYP was over 18 or aged 16 or over and no longer in school. The CNSS states that CAMHS is provided to all 0–18-year-olds, and while it has been acknowledged that many teams will not see CYP who are over 16 and out of school, all CAMHS in Scotland have been encouraged to see all CYP up to 18. Of the three rejected referrals for CYP who were 16 and no longer in school, two were for a recent suicide attempt. For one of these, the CYP was already being seen in adult mental health services. For the other, the RMG requested that the referrer re-direct to adult mental health services. One referral reported suicidal ideation with a concrete plan, and RMG requested re-referral to adult mental health services. It is possible that directing a referral to the inappropriate service, which then requires to be reviewed and redirected, adds an unnecessary time to accessing support for a suicidal CYP.

We conducted analyses to determine whether severity of suicidality impacted upon outcome. Chi-square analysis revealed that fewer referrals that mentioned passive suicidal ideation were accepted into the service than expected (190 vs. 202.6), and more that were for recent suicide attempts were accepted than expected (109 vs. 94.5) (Pearson’s chi-square = 33.03, Cramer’s *V* = 0.26, *p* < 0.001).

Of referrals which mentioned suicidality and for which referrals were available for review (*n* = 478), 208 (43.6%) did not include any evidence of risk assessment, 233 (48.7%) included a partial risk assessment, and 31 (6.5%) included a comprehensive risk assessment. A total of 324 (67.7%) included no evidence of safety planning, 116 (24.3%) included a partial safety plan, and 32 (6.7%) included a complete safety plan.

As we found a smaller number of boys to be referred for suicidality than girls, we conducted analyses to determine whether there were any differences by sex in the presence of risk assessment or safety planning and in outcomes of referral. Chi-square analysis revealed that girls were more likely than expected to have been given a comprehensive risk assessment (28 vs. 22.6), and boys were less likely than expected to have received this (four vs. 9.4), and this association was significant (Pearson’s chi-square = 4.73, Cramer’s *V* = 0.1, *p* = 0.03). There were no significant associations between sex and likelihood of safety planning or acceptance into CAMHS (*p* = 0.42).

## Discussion

Here we sought (1) to determine whether response to referrals for suicidality were appropriate in the context of the CNSS (2), to determine whether suicidal CYP were being missed, and, if so (3), to identify points of failure in a Scottish CAMHS. The majority of referrals for suicidality that were rejected represented appropriate decisions in the context of national policy. The majority of referrals for suicidal CYP did not include a risk assessment or safety plan. In three cases, the referrals that were made for CYP were over 16 and no longer in school, and they were redirected to adult mental health services. A number of CYPs’ referrals for passive suicidal ideation were rejected as they did not include evidence for serious mental ill health, and in most cases these were redirected to tiers 1 and 2 or other services. We argue in the following that we require changes to national service specifications that clarify what is meant by “suicidal behaviours”, where young people who present with suicidality without comorbid significant and impairing mental illness should be referred to if not CAMHS, the development and delivery of training and support in risk assessment and safety planning for referrers, and a review of the evidence concerning the ways in which girls and boys differ in their presentations, which may lead to fewer risk assessments for boys.

A total of 20.34% of referrals received by the Scottish CAMHS team over two 6-month periods included reference to suicidality. This is broadly consistent with the rates identified by (3) for two different Scottish Health Boards in 2019 (24%–26%). Small differences in rates of referrals mentioning suicidality across regions may reflect random fluctuations across a wider distribution, or it is possible that they are due to systemic differences in referral culture, levels of suicidality across regions, or coding differences across projects. It may also reflect the significant reduction in referrals mentioning suicidality between 2019 and 2022 that we identified in our sample. Further timepoints, however, must be assessed in order to determine whether this is a consistent trend and to determine whether there are broader shifts in referral patterns that explain this—for example, is the age profile of referrals shifting towards younger CYP? Or is the proportion of referrals for mental health difficulties other than suicidality increasing?

We sought to determine whether the outcomes of referrals that mentioned suicidality were appropriate in the Scottish policy context. Overall, we found that 82.4% of CYP whose referrals mentioned suicidality were offered CAMHS input. This is consistent with results from one CAMHS site audited by Gilmour et al. (2022) (3) and higher than their second site (82% and 31%). Differences such as these across regions operating under the same CNSS (4) policy context are perhaps unsurprising when there is a lack of clarity around the policy’s referral criteria (e.g., in the meaning of “suicidal behaviours”). Teams will inevitably be interpreting this differently. Suicidality can be divided into a motivational phase (i.e., experience of suicidal thoughts) and a volitional phase (i.e., suicidal action or behaviour) (5). Therefore, a conservative view of the CNSS could be that only CYP who have made suicide attempts (i.e., have taken action or exhibited suicidal behaviours) are eligible for CAMHS input. Alternatively, a broader view of suicidality which incorporates thoughts of suicide, with or without an intention to act on these thoughts, could also apply. It is likely, then, that different CAMHS teams are implementing different decision-making strategies about the appropriateness of tiers 3 and 4 levels of intervention for suicidality at different levels of severity. Greater clarity on the meaning of such terms is required, as is a service specification which acknowledges the evidence bases for most appropriate sources and types of support for CYP with suicidality at different levels of severity and with and without comorbid mental illness. We are gathering increasing evidence that, without this clarity, CAMHS teams across Scotland are making different decisions and CYP are receiving different responses to their suicidality depending upon where they live. Our evidence presented here, and our own anecdotal local clinical experience, is that CYP presenting with suicidality are being directed (or re-directed) to tier 2 supports (see information presented under “Disposal pathway” in **Table 3**). Locally, these are often third-sector organizations, and we acknowledge that the availability of these is a further source of variation across Scottish health boards. We need a Scotland-wide needs analysis on which service delivery is based, which specifies evidence-based responses to CYP with suicidality at all levels of severity, with and without comorbid mental health difficulties.

Of the 17.3% referrals that were rejected, the majority were rejected for appropriate reasons: the CYP was already being supported by CAMHS (15.5% of the rejected referrals), was already on the waiting list (27.8%), was not resident in the region (3.1%), had not provided consent (3.1%), or had insufficient information provided in the referral (4.1%). From this, we have learned that “rejection” can be appropriate and a majority of rejected referrals are due not to a failure to offer appropriate support by CAMHS but to inappropriate referrals. Writing and reviewing a referral, communication between CAMHS and the referrer, and onward referral to a more appropriate service are wasteful and may add delays to provision of care. This highlights the importance of better communication between CAMHS and referrers (6). This could include, for example, improve referral paperwork and national training co-designed by CAMHS clinicians and referrer stakeholders.

A total of 43.6% of referrals which mentioned suicidality did not include any risk assessment, and 67.7% did not include a safety plan. Without adequate information about risk, it is difficult for services such as CAMHS to adequately identify the appropriate support, and a CYP is potentially being added to a waiting list for inappropriate support without any thought being given to safety planning in the meantime. This is a further reason for greater communication between CAMHS and referrers to identify best practice and support for referrers of suicidal CYP as well as to feed into service delivery models which provide meaningful support for suicidal CYP at the point of referral. We recommend the development and delivery of national training in risk assessment and safety planning for referrers, again based on a national needs assessment and with consideration of sex differences in the ways in which suicidality may present in practice.

More referrals for girls mentioned suicidality than was expected and fewer for boys. There is evidence that boys and girls are responded to differently by mental health services (e.g (7).,); therefore, we carried out unplanned follow-up analyses to determine whether sex impacted upon outcome of referral and whether or not safety planning and risk assessment were conducted at referral. We found that boys were less likely than expected to receive safety planning and risk assessment at referral, whereas the pattern was reversed for girls. There was no impact of sex on outcome of referral. There is an evidence base which shows the “gender paradox” of suicide such that men are more likely to die by suicide while women report higher rates of self-harm and suicidal thoughts and attempts (8). Further research is required, however, to determine whether boys are truly experiencing less suicidality than girls or are instead presenting or describing different symptoms of suicidality, are less likely to be asked about suicidality by referrers, or are less likely to disclose suicidality.

We identified a potential point of failure around the age limits at which CAMHS will offer input to CYP in the current study site. The CNSS (2) states that CAMHS should offer support to all CYP aged 0–18. Many CAMHS teams including the study site, however, add a caveat that CYP aged 16 or over who are not registered with school will not be supported and are instead seen within adult mental health services. This fits with the Age of Legal Capacity Act (1991) (9), in which those over 16 are considered to hold legal capacity, and is a source of variability across CAMHS teams in Scotland and the UK (e.g., 10). Of the three rejected referrals for CYP who were 16 and no longer in school, one was already being seen in adult mental health services, and CAMHS requested that the referrer redirect the other two referrals to adult mental health services. One of these was for a recent suicide attempt and the other for suicidal ideation with a concrete plan. By referrals being inappropriately directed to CAMHS rather than adult mental health services and by CAMHS not accepting the referrals due to the CYP no longer being in school despite being under 18, it is possible that processes caused an avoidable delay in support for CYP at risk of suicide. We argue that better linkage between child and adult mental health services, including shared referral management for those within overlapping age ranges, is required.

There were missing data on all variables; however, our sample size was large enough for us to have confidence that we have provided an accurate description of, at the very least, broad patterns in our service’s response to referrals that mention suicidality. We have not followed up CYP to understand their journeys following rejection or acceptance from CAMHS. Finally, this is a case study of a single health board in a Scottish policy context. We are reassured, however, by broad similarities between our results and those of previous studies (3) and believe that our results and recommendations are of relevance to all UK CAMHS services using a tiered system. We do not know how referral information is gathered and whether young people are routinely asked about suicidality. It is possible, therefore, that a proportion of referrals in which suicidality is not mentioned are for young people experiencing suicidal thoughts or actions.

Future research should seek to explore the experience of CYP following disclosure of suicidality. We know from recent work (e.g (11)., and (12)) that many CYP feel CAMHS staff can be invalidating and focus on risk assessment rather than supporting the CYP to feel better. They also tell us that, when they seek support, it is often not available or is not what they actually need (13). Better understanding these experiences should be the groundwork of service improvement and delivery.

In conclusion, our findings highlight the importance of clearer communication between CAMHS and referrers to reduce inappropriate referrals, to improve pathways for re-direction to other services, and to support referrers to include risk assessment and safety planning. We identified a lack of clarity in national guidelines around the meaning of “suicidal behaviours” and the necessity for evidence of co-morbid mental illness. Future work should assess the national picture of suicidality in CYP and base service delivery upon this and evidence-based responses to and interventions for suicidality. We should seek to understand the experiences of CYP and to develop models of service delivery that meet the needs of CYP expressing all levels of suicidality, with and without comorbid mental ill health.

## Data Availability

The raw data supporting the conclusions of this article will be made available by the authors, without undue reservation.

## References

[1] Public Health Scotland . Suicide Among Young People in Scotland: A Report From the Scottish Suicide Information Database (2022). Available online at: https://www.publichealthscotland.scot/publications/scottish-suicide-information-database/suicide-among-young-people-in-scotland-a-report-from-the-scottish-suicide-information-database-6-september-2022/ (Accessed March 13, 2026).

[2] Scottish Government . Child and Adolescent Mental Health Services: National Service Specification (2020). Available online at: https://www.gov.scot/publications/child-adolescent-mental-health-services-camhs-nhs-scotland-national-service-specification/ (Accessed March 13, 2026).

[3] Gilmour L Best C Duncan E Maxwell M . Characteristics and outcomes of referrals to CAMHS for children who are thinking about or attempted suicide: A retrospective cohort study in two Scottish CAMHS. Front Psychiatry. (2022) 13:914479. doi: 10.3389/fpsyt.2022.914479 36117645 PMC9477378

[4] Posner K Brent D Lucas C Gould M Stanley B Brown G . Columbia-Suicide Severity Rating Scale (C-Ssrs) Vol. 10. . New York, NY: Columbia University Medical Center (2008). p. 2008.

[5] O'Connor RC Kirtley OJ . The integrated motivational–volitional model of suicidal behaviour. Philos Trans R Soc B Biol Sci. (2018) 373:20170268. 10.1098/rstb.2017.0268 PMC6053985 30012735

[6] Smith J Kyle RG Daniel B Hubbard G . Patterns of referral and waiting times for specialist Child and Adolescent Mental Health Services. Child Adolesc Ment Health. (2018) 23:41–9. doi: 10.1111/camh.12207 32677372

[7] Ball WP Black C Gordon S Ostrovska B Paranjothy S Rasalam A . Inequalities in children’s mental health care: analysis of routinely collected data on prescribing and referrals to secondary care. BMC Psychiatry. (2023) 23:22. doi: 10.1186/s12888-022-04438-5 36627578 PMC9831880

[8] Miranda-Mendizabal A Castellví P Parés-Badell O Alayo I Almenara J Alonso I . Gender differences in suicidal behavior in adolescents and young adults: systematic review and meta-analysis of longitudinal studies. Int J Public Health. (2019) 64:265. doi: 10.1007/s00038-018-1196-1 30635683 PMC6439147

[9] Scottish Government . Adults With Incapacity Act (2000) (2000). Available online at: https://www.legislation.gov.uk/asp/2000/4/contents (Accessed March 13, 2026).

[10] MIND . At What Age Will I Leave CAMHS? (2024). Available online at: https://www.mind.org.uk/for-young-people/camhs/when-will-i-leave-camhs/#:~:text=Most%20CAMHS%20offer%20support%20to,you%20until%20you're%2025 (Accessed March 13, 2026).

[11] Foster M Frith H John M . ‘I'm still su! c! dal when you're done with the paperwork’: an inductive framework thematic analysis of# camhs on TikTok. J Child Psychol Psychiatry. (2024) 65:1258–69. 10.1111/jcpp.14002 38724448

[12] Gilmour L Ring N Maxwell M . The views and experiences of suicidal children and young people of mental health support services: a meta‐ethnography. Child Adolesc Ment Health. (2019) 24:217–29. doi: 10.1111/camh.12328 32677214

[13] Samaritans . Loneliness, Suicide and Young People (2019). Available online at: https://media.samaritans.org/documents/loneliness-suicide-young-people-jan-2019.pdf (Accessed March 13, 2026).

